# Long-term follow-up results of ruxolitinib as salvage therapy for chronic graft-versus-host disease

**DOI:** 10.1016/j.htct.2025.103835

**Published:** 2025-05-11

**Authors:** Neslihan Mandaci Sanli, Esen Karakuş

**Affiliations:** a Erciyes University Faculty of Medicine, Department of Hematology and Bone Marrow Transplant Center, 38039, Kayseri, Turkey; b Erciyes University Faculty of Medicine, 38280 ,Kayseri, Turkey

**Keywords:** Chronic graft-versus-host disease, Ruxolitinib, Allogeneic hematopoietic stem cell transplantation, Stem cell transplantation

## Abstract

**Introduction:**

Chronic graft-versus-host disease poses a significant challenge after allogeneic hematopoietic stem cell transplantation with initial treatment often relying on high-dose steroids. However, managing steroid-refractory disease remains daunting. Recent insights into the mechanisms have unveiled new treatment targets, with ruxolitinib, a selective JAK1/2 inhibitor, emerging as a promising and safe therapy for chronic graft-versus-host disease patients.

**Methods:**

This retrospective study describes the long-term outcomes of 23 chronic graft-versus-host disease patients treated with ruxolitinib.

**Results:**

Most patients presented with severe chronic graft-versus-host disease (15/23; 65.2%). The overall response rate was 78.3% (18/23) after a median treatment duration of four weeks, with 55.6% (10/18) achieving complete response. At follow-up, 13 of the 18 responders (72.2%) sustained complete remission. Patients had a median of two previous lines of therapy, with a median follow-up of 14 months (range: 2–46 months) after starting ruxolitinib. Of the patients who were responsive to ruxolitinib, median follow-up extended to 26.5 months. Notably, for the patients who were responsive to ruxolitinib, the 1-year, 2-year, and 3-year overall survival was 83.3% (95% CI: 64.2%-102%), 56.1% (95% CI: 30.1%-80.9%), and 33.3% (95% CI: 9.2%-57.4%), respectively. Malignancy relapse occurred in 17.4% (4/23) of patients, with 34.7% (8/23) experiencing cytopenias, albeit mostly mild. Reactivation rates for cytomegalovirus were nil.

**Conclusion:**

The long-term follow-up in this study supports ruxolitinib as an effective salvage therapy for chronic graft-versus-host disease with a 78.3% overall response rate and 55.6% complete remission rate. However, large prospective studies are warranted to validate these findings

## Introduction

Allogeneic hematopoietic stem cell transplantation (allo-HSCT) is a pivotal treatment for patients afflicted with hematological malignancies and non-malignant diseases.^1^ The success of allo-HSCT hinges on two primary factors: the management of transplant-related complications and disease relapse.^2^ Notably, chronic graft-versus-host disease (cGvHD) stands out as a significant contributor to procedural morbidity and relapse-free mortality, arising in 35–70% of allo-HSCT recipients.^1^^,^^2^ cGvHD is a multisystem clinical syndrome caused by donor-mediated immune reactions in HSCT recipients^3^ with corticosteroids being the mainstay treatment. However, approximately half of cGvHD patients exhibit resistance to corticosteroid therapy, and more than half require second-line treatment within two years.^4^ For cGvHD, second-line therapies include calcineurin inhibitors, extracorporeal photopheresis, ibrutinib, Janus kinases (JAK) inhibitors, mycophenolate mofetil, rituximab, mammalian target of rapamycin inhibitors, pentostatin, proteasome inhibitors, and tyrosine kinase inhibitors.^5^^,^^6^

Among the array of treatments or interventions available, a consensus has yet to be reached regarding the optimal salvage therapy for steroid-refractory (SR)-cGvHD. For years, the intricate pathophysiology of cGvHD has posed a formidable challenge in its management.^7^ However, advancements in understanding the underlying pathways have paved the way for novel treatment modalities targeting these mechanisms.^7^^,^^8^ Among these, interventions aiming at kinase activity have emerged as promising strategies, showing encouraging outcomes in both preclinical models and clinical trials.^9^

JAK1 and 2 (JAK1/2) have garnered significant attention in GvHD research due to their pivotal roles in cytokine production and activation of inflammatory cells.^10^ Ruxolitinib, a selective oral inhibitor targeting JAK1/2-signal transducer and activator of transcription (STAT) signaling, holds promise in mitigating these pathways.^11^ JAKs facilitate signaling from various cytokine receptor family members and play a critical role in the inflammatory cascade, leading to tissue damage and fibrosis in cGvHD.^10^ By targeting JAK1/2 signaling, inhibitors like ruxolitinib may impede multiple facets of T-cell activation, including donor T-cell expansion, cytokine production, and B-cell differentiation while promoting regulatory T-cell (Treg) function.^9^^,^^12^ This multifaceted inhibition could potentially alleviate disease severity by suppressing proinflammatory cytokines.^9^

Moreover, unlike conventional immunosuppressive agents that primarily affect T-cell function, ruxolitinib has been shown to disrupt dendritic cell differentiation, maturation, and cytokine production, potentially enhancing its efficacy against GvHD.^12^^,^^13^ Building on this foundation, Zeiser et al.^14^ documented successful ruxolitinib therapy for human GvHD in 2015. A retrospective review of ruxolitinib use in Chinese patients with GvHD revealed an overall response rate (ORR) of 82.1% for cGvHD.^15^ Another study assessing the long-term outcomes of ruxolitinib treatment in 35 patients with SR-cGvHD documented an ORR of 89%, with 26% achieving a complete response (CR).^16^

Recently, Zeiser et al.^17^ presented findings from a prospective study that compared ruxolitinib with the current optimal treatment, yielding a noteworthy best ORR of 76%. This study holds significance as it provides a prospective evaluation of the efficacy of ruxolitinib. Following this trial, in September 2021, the Food and Drug Administration (FDA) approved ruxolitinib to treat patients aged 12 years and above with cGvHD who have experienced treatment failure with one or two lines of systemic therapy.^6^

Retrospective studies assessing the effectiveness of ruxolitinib in SR-cGvHD often need more median follow-up durations, hampering accurate assessments of response duration and long-term outcomes.^17^, ^18^, ^19^, ^20^, ^21^, ^22^ Hence, investigations with extended follow-up periods are crucial for comprehensive understanding. This paper presents the long-term outcomes of ruxolitinib treatment in 23 patients with cGvHD.

## Materials and methods

In this retrospective analysis conducted at a single center, 23 recipients of allo-HSCT with cGvHD who underwent salvage therapy with ruxolitinib between December 2018 and December 2022 were examined. The initial ruxolitinib dosage (5 or 10 mg twice daily) was determined based on individual hematological parameters. Basic transplant-related information was gathered and is summarized in Table 1. Additionally, the time intervals from transplantation to the onset of cGvHD and from cGvHD onset to the initiation of ruxolitinib treatment were recorded.

**Table 1:** Patient characteristics at the start of ruxolitinib therapy.

Variable	Result
Patients – n	23
Age, years - median (range)	46 (30–67)
Gender (male/female) - n (%)	10 (43.5)/13 (56.5)
Diagnosis - n (%)	
Acute myelogenous leukemia	13 (56.5)
Acute lymphoblastic leukemia	6 (26)
Myelodysplastic syndrome	1 (4.3)
Lymphoma	3 (14)
Conditioning regimen - n (%)	
Myeloablative	17 (73.9)
Reduced intensity or nonmyeloablative	6 (26.1)
Donor - n (%)	
Matched related donor	20 (87)
Unrelated donor	1 (4.3)
Haploidentical donor	2 (8.7)
CMV serostatus	
R-/D-	5 (21.7)
R-/*D*+	3 (13)
*R*+ /D-	4 (17.4)
*R*+ /*D*+	11 (47.8)
GvHD prophylaxis - n (%)	
Cyclosporine + Mtx	20 (87)
Cyclosporine + Mtx + ATG	1 (4.3)
PT-Cy + Cyclosporine + MMF	2 (8.7)
cGvHD severity	
Moderate	8 (34.8)
Severe	15 (65.2)
Organ involvement of cGvHD - median (range)	1 (1–3)
Previous therapies before ruxolitinib - median (range)	2 (2–5)
Time from cGvHD to start of ruxolitinib treatment (days) - median (range)	40 (60–180)
Duration of ruxolitinib treatment (months) - median (range)	14 (2–46)
Follow-up after ruxolitinib treatment initiation (months) - median (range)	14 (2–46)
Time to response (weeks) - median (range)	4 (1–21)

cGvHD, chronic graft-versus-host disease; HLA, human leukocyte antigen; PT-Cy, post-transplant endoxan; MTX, methotrexate; MMF, mycophenolate mofetil; ATG, anti-thymocyte globulin; *R*+, recipient CMV positive; R-, recipient CMV negative; d-, donor CMV negative; *D*+, donor CMV positive.

Prior to commencing ruxolitinib therapy, the affected organ sites were stratified and cGvHD was graded as per the National Institutes of Health (NIH) 2015 criteria.^23^ Response assessment adhered to NIH criteria, delineating responses as CR, partial response (PR), or lack of response (unchanged, mixed response, or progression). CR signified the complete resolution of all disease manifestations across all involved organs or sites, whereas PR indicated improvement in at least one organ or site without progression. Lack of response encompassed disease progression in any organ, site, or outcomes not meeting CR or PR criteria. The ORR is the proportion of patients achieving CR and PR. Overall survival (OS) was determined as the time elapsed from the initiation of ruxolitinib treatment to the last follow-up or death. This study diligently documented prevalent adverse events linked with ruxolitinib, including cytopenias and infections, and categorized toxicities based on the grading of the National Cancer Institute Common Terminology Criteria for Adverse Events.

Approval for the study was granted by the Erciyes University Faculty of Medicine Ethics Committee (Date: 26–04–2023, Decision No: 2023/311). All procedures adhered to ethical guidelines and the principles outlined in the Helsinki Declaration.

Patient characteristics are summarized using descriptive statistics. OS was determined using the Kaplan-Meier method. Descriptive analyses are presented as numbers (n), percentages (%), and 95% confidence intervals (95% CIs).

## Results

The cohort of this study consisted of 23 patients who underwent salvage therapy with ruxolitinib; their characteristics are outlined in Table 1. The median age was 46 years (range: 30–67 years), with a male-to-female ratio of 10/13 (43.5/56.5%). The most prevalent diagnoses were acute myeloid leukemia (AML) in 13 patients (56.5%) and acute lymphoblastic leukemia (ALL) in six patients (26%). Graft sources included human leukocyte antigen (HLA)-matched related donors in 20 patients (87%), HLA-matched unrelated donors in one patient (4.3%), and HLA haploidentical donors in two patients (8.7%). The majority of patients underwent myeloablative conditioning regimens (73.9%).

Regarding cGvHD severity, eight patients (34.8%) had moderate cGvHD, while 15 patients (65.2%) had severe cGvHD. The affected organs included the liver in 52.2% (12/23) of patients, lung in 8.7% (2/23), oral mucosa in 30.4% (7/23), gastrointestinal system in 13% (3/23), and skin in 43.5% (10/23). The median number of prior therapy lines was two (range: 2–5), with ruxolitinib administered as the third line in ten patients, fourth line in seven patients, fifth line in three patients, and sixth line in three patients.

The median duration from the onset of cGvHD to the commencement of ruxolitinib therapy was 60 days (range: 40–180 days), with a median response time of four weeks (range: 1–21 weeks) after initiation of ruxolitinib. Of the 23 patients, 18 exhibited a response to ruxolitinib, resulting in an ORR of 78.3%. Of these responders, the majority (55.6%) achieved CR at a median of four weeks into treatment. Eight patients (45.4%) achieved PR, with three of them switching to CR during follow-up, culminating in a total of 13 patients (72.2%) reaching CR during ruxolitinib therapy. Of the patients who were responsive to ruxolitinib, prednisone was successfully tapered to physiologic doses in three patients (16.8%) and discontinued in 15 patients (83.2%) at a median of 51 days (range: 10–90 days) after ruxolitinib initiation.

Five patients (21.7%) exhibited no response to ruxolitinib, as outlined in Table 2. All five patients presented with severe cGvHD; one had pulmonary involvement, resulting in significant sequelae and pleuroparenchymal fibroelastosis, three had mouth involvement and all five patients manifested sclerotic changes with skin involvement. Of the patients who were not responsive to ruxolitinib, two patients experienced relapse and subsequent mortality, one within the second month of ruxolitinib treatment and the other within the third month of ruxolitinib treatment.

**Table 2:** Details of patients.

#	Age	Gender	cGvHD onset^a^	Donor	Global cGvHD score	Involved sites	Prior therapies^b^	Day for RUX^c^	Response to RUX^d^	Duration m^e^	Response to RUX^f^	Status/IS	Follow-up, m	Stopping RUX^g^
1	61	female	9	MRD	mild	liver	Steroids, CSP	45	PR	15	PR	Death from covid-19/RUX, CSP	15	–
2	38	female	5	MRD	mild	liver	Steroids, CSP, MSC, ibrutinib	180	CR	17	CR	Alive/-	31	+
3	45	female	4	MUD	severe	liver	Steroids, CSP	45	PR	29	CR	Death from covid19/RUX, CSP	29	–
4	46	female	4	MRD	severe	skin, mouth	Steroids, CSP	120	PR	27	CR	Alive/-	36	+
5	46	male	4	MRD	severe	liver	Steroids, CSP	45	CR	36	CR	Alive/-	41	+
6	67	female	14	MRD	severe	mouth	Steroids, CSP	90	PR	24	PR	Alive/-	31	+
7	30	female	4	HID	mild	skin, gut	Steroids, CSP, MMF, MSC, ECP	60	PR	32	CR	Alive/RUX	38	–
8	41	male	11	MRD	severe	lung	Steroids, CSP, MMF	90	PR	24	PR	Alive/-	37	+
9	57	male	7	MRD	mild	liver	Steroids, CSP, MMF, ECP	45	CR	33	CR	Alive/-	39	+
10	57	male	9	MRD	severe	liver	Steroids, CSP, MMF	120	PR	46	PR	Alive/RUX	46	–
11	51	female	7	MRD	severe	Lung skin, mouth	Steroids, CSP, MMF	90	Lack of response lung: unchanged others: PR	14	lung: Lack of response others: PR	Death from refractory cGvHD/RUX, Steroid, CSP, MMF	14	–
12	55	female	4	MRD	severe	skin	Steroids, CSP	40	Lack of response	3	Lack of response	Death from relapse/Ruxolitinib, CSP	3	–
13	37	female	10	MRD	severe	liver	Steroids, CSP, MMF, MSC, ECP	120	CR	23	CR	Death from relapse/RUX	23	–
14	32	male	9	MRD	severe	Skin mouth	Steroids, CSP, imatinib, rituximab, ECP	150	Lack of response	2	Lack of response	Death from relapse/RUX, ECP	2	–
15	59	female	20	MRD	severe	skin	Steroids, CSP	110	Lack of response	5	Lack of response	Death from covid-19/RUX, CSP, ECP	5	–
16	54	male	5	MRD	mild	Liver mouth	Steroids, CSP, MMF	60	CR	2	CR	Death from relapse/RUX	2	–
17	32	male	3	MRD	severe	Liver skin	Steroids, CSP, İmatinib	90	CR	12	CR	Alive/RUX	12	–
18	43	male	4	MRD	mild	Liver skin	Steroids, CSP	60	CR	13	CR	Alive/RUX	13	–
19	33	female	4	MRD	mild	skin	Steroids, CSP, MMF	60	PR	6	PR	Alive/RUX, MMF	6	–
20	48	female	4	MRD	severe	gut	Steroids, CSP, MSC, ECP	50	CR	12	CR	Alive/RUX	12	–
21	63	female	11	MRD	mild	Liver mouth	Steroids CSP, ECP	45	CR	10	CR	Alive/RUX	10	–
22	38	male	4	MRD	severe	Skin mouth	Steroids, CSP	60	Lack of response	12	Lack of response	Alive/RUX, ECP, MMF	12	–
23	56	male	3	HID	severe	Liver gut	Steroids, MMF	40	CR	14	CR	Alive/RUX	14	–

MUD, matched-unrelated donor; MRD, matched-related donor; HID, haploidentical donor; RUX, Ruxolitinib; PR, partial response; CR, complete response; cGvHD, chronic graft-versus-host disease.

a posttransplant month, the onset of GvHD attack in which ruxolitinib treatment was started.

b Therapies administered in the treatment of cGvHD before RUX.

c day from onset of cGvHD to initiation of ruxolitinib treatment.

d response after a median 4 weeks of ruxolitinib treatment; m:month.

e Total duration of ruxolitinib administration.

f response to ruxolitinib treatment at the last follow-up.

g Whether or not RUX was discontinued after RUX treatment MMF, mycophenolate mofetil; CSP, cyclosporine A; MSC, mesenchymal stem cells; ECP, extracorporeal photopheresis; IS, Immunosuppressants administered for cGvHD treatment at the last follow-up. Patient 1 diagnosed with mild liver cGvHD achieved a PR after a median of four weeks of ruxolitinib treatment. The patient passed away from COVID-19 while the PR continued after 15 months of treatment. Follow-up period was 15 months. Patient 2 diagnosed with mild liver cGvHD achieved a CR after a median of four weeks of treatment. The CR persisted for 17 months of treatment and ruxolitinib was discontinued. No recurrence of cGvHD was observed during 14 months of drug-free follow-up. Follow-up period was 31 months. Patient 3 diagnosed with severe liver cGvHD achieved a PR after a median of four weeks of treatment. The response converted to a CR by the 6th month of treatment. The patient passed away from COVID-19 while the CR continued after 29 weeks of treatment. Follow-up period was 29 months Patient 4 diagnosed with severe skin and mouth cGvHD achieved a PR after a median of four weeks of treatment. The response converted to a CR within the first year of treatment. CR was maintained after 27 months of treatment, and ruxolitinib was discontinued. No cGvHD recurrence was observed during 9 months of drug-free follow-up. Follow-up period was 36 months Patient 5 diagnosed with severe liver cGvHD achieved a CR after a median of four weeks of treatment. The CR was sustained after 36 months of treatment, and ruxolitinib was discontinued. No cGvHD recurrence was observed during five months of drug-free follow-up. Follow-up period was 41 months. Patient 6 diagnosed with severe mouth cGvHD achieved a PR after four weeks of treatment. The PR was maintained after 24 months of treatment, and ruxolitinib was discontinued. No cGvHD recurrence was observed during seven months of drug-free follow-up. Follow-up period was 31 months. Patient 7 diagnosed with mild skin and gut cGvHD achieved a PR after a median of four weeks of treatment. The response converted to a CR within the first year of treatment and maintained after 32 months of treatment, even after ruxolitinib was discontinued. No cGvHD recurrence was observed during six months of drug-free follow-up. Follow-up period was 38 months. Patient 8 diagnosed with severe lung cGvHD achieved a PR after a median of four weeks of treatment. The PR continued after 24 months of treatment, and ruxolitinib was discontinued. No cGvHD recurrence was observed during 13 months of drug-free follow-up. Follow-up period was 37 months. Patient 9 diagnosed with mild liver cGvHD achieved a CR after a median of four weeks of treatment. The CR continued after 33 months of treatment, and ruxolitinib was discontinued. No cGvHD recurrence was observed during six months of drug-free follow-up. Follow-up period was 39 months. Patient 10 diagnosed with severe liver cGvHD achieved a PR after a median of four weeks of treatment. The PR continued after 46 months of treatment, and the patient remains on medication. Follow-up period was 46 months. Patient 11 diagnosed with severe lung, skin, and mouth cGvHD showed no response after a median of four weeks of treatment. The lack of response persisted after 14 months (lung: unchanged, skin: PR, and mouth: PR) and the patient eventually passed away due to cGvHD. Follow-up period was 14 months. Patient 12 diagnosed with severe skin cGvHD showed no response after a median of four weeks of treatment. The lack of response persisted after three months of treatment, and the patient passed away due to a relapse of the primary disease. Follow-up period was three months. Patient 13 diagnosed with severe liver cGvHD achieved a CR after a median of four weeks of treatment. The CR continued after 23 months of treatment, but the patient passed away due to a relapse of the primary disease. Follow-up period was 24 months. Patient 14 diagnosed with severe skin and mouth cGvHD showed no response after a median of four weeks of treatment. The lack of response continued after two months of treatment and the patient passed away due to a relapse of the primary disease. Follow-up period was two months. Patient 15 diagnosed with severe skin cGvHD showed no response after a median of four weeks of treatment. The lack of response persisted after five months, and the patient passed away due to COVID-19. Follow-up period was five months. Patient 16 diagnosed with mild liver and mouth cGvHD achieved a CR after a median of four weeks of treatment. The CR continued after two months of treatment, but the patient passed away due to a recurrence of the primary disease. Follow-up period was two months. Patient 17 diagnosed with severe liver and skin cGvHD achieved a CR after a median of four weeks of treatment. The CR continued at the end of 12 months of treatment. Follow-up period was 12 months. Patient 18 diagnosed with mild liver and skin cGvHD achieved a CR after a median of four weeks of treatment. The CR continued at the end of 13 months of treatment. Follow-up period was 13 months. Patient 19 diagnosed with mild skin cGvHD achieved a PR after a median of four weeks of treatment. The PR continued at the end of six months of treatment. Follow-up period was six months. Patient 20 diagnosed with severe gut cGvHD achieved a CR after a median of four weeks of treatment. The CR continued after 12 months of treatment. Follow-up period was 12 months. Patient 21 diagnosed with mild liver and mouth cGvHD achieved a CR after a median of four weeks of treatment. The CR continued after ten months of treatment. Follow-up period was ten months. Patient 22 diagnosed with severe skin and mouth cGvHD showed no response after a median of four weeks of treatment. The lack of response persisted after 12 months of treatment. Follow-up period was 12 months. Patient 23 diagnosed with severe gut and liver cGvHD achieved a CR after a median of four weeks of treatment. The CR continued after 14 months of treatment. Follow-up period was 14 months.

During follow-up, 15 patients (85.2%) remained alive, while eight patients (34.8%) died. The causes of death included coronavirus disease-2019 (COVID-19) in three patients, refractory cGvHD in one patient, and relapse in four patients.

**Figure 1:**
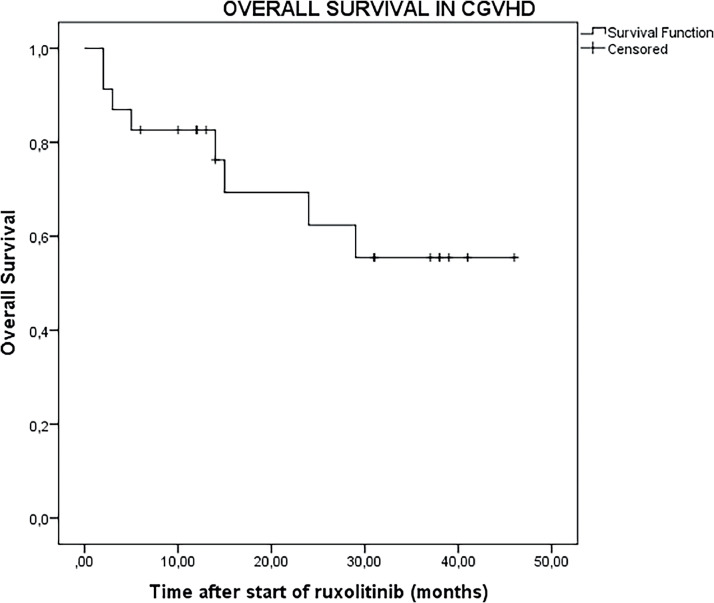
Kaplan-Meier curve showing overall survival for all patients after initiation of ruxolitinib treatment.

**Figure 2:**
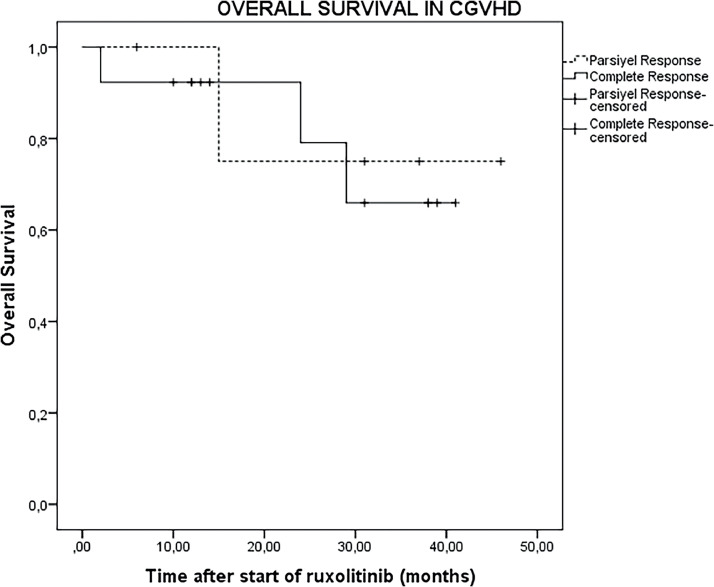
Kaplan-Meier curve showing overall survival for patients who responded to ruxolitinib treatment.

The median follow-up duration after initiation of ruxolitinib was 14 months (range: 2–46 months) for all 23 patients and extended to 26 months (range: 2–46 months) for the 18 patients who were responsive to ruxolitinib. In the entire cohort of 23 patients, the OS was 73.9% (95% CI: 54.5–93.3%) at 1 year, 43.4% (95% CI: 21.6–65.4%) at 2 years, and 26.1% (95% CI: 6.6–45.5%) at 3 years (Figure 1). For the 18 patients who were responsive to ruxolitinib, the OS was 83.3% (95% CI: 64.2–102.4%) at one year, 56.1% (95% CI: 30.1–80.9%) at two years, and 33.3% (95% CI: 9.2–57.4%) at three years (Figure 2).

The median treatment duration spanned 14 months (range: 2–46 months) for all 23 patients and 20 months (range: 2–46 months) for the 18 patients who were responsive to ruxolitinib. After the follow-up period, of the patients who were responsive to ruxolitinib, nine (50%) relied solely on ruxolitinib as an immunosuppressive agent and maintained either PR (*n* = 2) or CR (*n* = 7), while three patients (16.7%) supplemented ruxolitinib with additional immunosuppressants (2 in CR and 1 in PR). Six patients (33.3%) discontinued ruxolitinib upon achieving sustained response, with a median treatment duration of 25.5 months (range: 17–36 months). Of these, four attained CR, and two met the criteria for PR. In cases of PR, residual cGvHD involvement was considered, and no further benefit was anticipated from maintaining the drug. Notably, cGvHD relapse was absent within a median of nine months (range: 5–14 months) following drug discontinuation.

### Adverse events

Table 3 shows the adverse events documented during ruxolitinib treatment. Hematologic toxicities were prevalent within this cohort, with eight patients (34.7%) experiencing cytopenias, including Grade 3 anemia in one patient and Grade 3 thrombocytopenia in another. Dose reduction resolved the issue in both cases of Grade 3 cytopenias, while in six patients, no alterations were made to avoid compromising the clinical benefits of the drug, with close monitoring for cytopenia-related symptoms.

**Table 3:** Adverse events (n = 19)/.

Event	n (%)
Infections	
Pneumonia and herpes zoster	1 (5.3)
Peripheral edema	2 (10.5)
CMV colitis	1 (5.3)
Vomiting	2 (10.5)
Severe cytopenia (Grade 3 and 4)	
Thrombocytopenia	1 (5.3)
Mild cytopenia (Grade 1 and 2)	
Neutropenia	2 (10.5)
Malignancy relapse	1 (5.3)

Throughout the follow-up period on ruxolitinib, bacterial infections affected 13% of patients (3/23), while viral infections affected 30.4% (7/23). Of the viral infections, there were two cases (8.7%) of herpes zoster, one case (4.3%) of human polyomavirus 1 (BK) virus, three cases (13%) of COVID-19, and one case (4.3%) had herpes simplex with oral lesions. Notably, severe BK-viral hemorrhagic cystitis was not observed, and no fungal events were diagnosed among patients undergoing cGvHD treatment.

No instances of cytomegalovirus (CMV) reactivation were detected in the 23 patients, suggesting that ruxolitinib may not significantly elevate the risk of CMV reactivation. Plasma CMV polymerase chain reaction (PCR) monitoring was conducted for all recipients.

Relapse of the underlying malignancy occurred in four patients (17.4%), with two being non-responsive to ruxolitinib. Of the patients who were responsive to ruxolitinib, two (11.1%) experienced relapses, one with refractory AML and the other with ALL. Both patients were on ruxolitinib at the time of relapse (approximately two months and 23 months, respectively), having achieved CR of cGvHD.

## Discussion

CGvHD remains the primary long-term complication after allo-HSCT, yet significant transformations have unfolded over the past decade. Novel strategies for managing cGvHD have shifted from broad, protracted immunosuppression with high-dose corticosteroids to therapies pinpointing specific mechanistic pathways relevant to cGvHD pathophysiology. By inhibiting JAK1/2, ruxolitinib addresses various facets of the immune response implicated in cGvHD, including allogeneic T cell proliferation and inflammatory cytokine generation.^9^^,^^24^^,^^25^ The favorable clinical outcomes of ruxolitinib in refractory cGvHD were initially highlighted by Zeiser et al.^14^ in 2015, with subsequent retrospective studies consistently corroborating its efficacy. Notably, the REACH3 trial has recently furnished robust evidence further advocating the utilization of ruxolitinib in this setting.^15^, ^16^, ^17^

In the current investigation, significant responses to ruxolitinib treatment were observed in cases of moderate and severe cGvHD. The analysis of the present study revealed an ORR of 78.3% after a median treatment duration of four weeks, with the majority of responses being CR (55.6%). These findings closely parallel those reported by Ferreira et al.^16^ in 2021, who conducted a long-term follow-up study of ruxolitinib in 35 cGvHD patients, demonstrating an ORR of 89% (with CR accounting for 26%) after a similar median treatment duration. Similarly, Wu et al.^27^ reported an ORR of 70.7% in 41 cGvHD patients treated with ruxolitinib. Furthermore, a Phase 3 randomized controlled study showcased favorable outcomes for ruxolitinib in cGvHD compared to the best available treatment, with an ORR of 50% versus 26% at Week 24.^17^ Notably, the current patient cohort exhibited a higher proportion of severe cGvHD cases (65.2%) compared to the REACH3 study (59%) and demonstrated a superior ORR (78.3%).

In the systematic review and meta-analysis conducted by Zang et al.,^5^ the ORR for cGvHD was documented as 73.1%. Additionally, a meta-analysis encompassing 26 studies investigating ruxolitinib in SR-cGvHD reported an ORR of 0.78 (95% CI: 0.74–0.81) at any time, with a two-year OS of 75.3% (95% CI: 68.0–82.7%).^4^ Examination of ORRs across studies focusing on ruxolitinib treatment for cGvHD reveals a wide range, varying from 45% to 89%.^16^^,^^20^^,^^21^^,^^28^

While the majority of patients in the studies by Ferrari et al.^16^ and Abedin et al.^22^ presented with moderate cGvHD, the current study predominantly included patients with severe cGvHD (65.2%). Consequently, achieving a high ORR in severe cGvHD patients is a significant outcome. Moreover, the majority of patients were responsive to ruxolitinib in this study achieving a CR rate of 72.2% at follow-up, representing the highest CR rate reported to date, whereas lower CR rates ranging from 3.5% to 36.6% were reported in other studies.^16^^,^^21^^,^^27^, ^28^, ^29^

Long-term follow-up reports of ruxolitinib treatment in cGvHD patients are largely confined to small retrospective analyses, with the majority of studies featuring a short-term follow-up ranging from 12 to 19 months.^18^, ^19^, ^20^, ^21^, ^22^ Moisev et al.^29^ documented a median follow-up time of 28 months and a median ruxolitinib duration of 23 months, reporting a one-year OS rate of 81%. In another study, Ferreira et al.^16^ reported a median follow-up of 43 months in 35 cGvHD patients. The present study contributes to this limited pool as one of the few investigations providing long-term follow-up data on ruxolitinib treatment in cGvHD patients.^16^, ^26^^,^^27^^,^^29^ In this study, the median follow-up duration after the initiation of ruxolitinib was 14 months for all 23 patients and 20.5 months for the 18 patients who were responsive to ruxolitinib. Of the patients who were responsive to ruxolitinib, 33.3% discontinued the drug, 50% received ruxolitinib as the sole immunosuppressive therapy, and no cGvHD relapse was observed. On the other hand, the study of Ferreira et al.^16^ reported that 15 patients had discontinued the drug, with only 22% receiving ruxolitinib as the sole immunosuppressive therapy.

In existing literature, studies have reported rates of steroid dose reduction to physiological levels or discontinuation of prednisone ranging from 57 to 89%.^16^^,^^19^^,^^21^ The primary objective in treating cGvHD is to alleviate the adverse effects associated with steroids and significantly improving the patient's quality of life by discontinuing steroids as early as possible. In the current study, all the patients who were responsive to ruxolitinib successfully reduced their steroid dose or discontinued it altogether. Our steroid discontinuation rate (83.2%) closely mirrors that reported by Ferreira et al.^16^ (81%), likely reflecting the high CR rate (55.6%) we achieved.

The optimal duration of ruxolitinib use in responsive patients, particularly after achieving CR, remains uncertain. Notably, the heightened immunosuppression resulting from the mechanism of action of ruxolitinib may increase the risk of relapse of the underlying malignancy.^13^ Only two relapses (11.1%) were observed in this study, both of which responded to ruxolitinib treatment. One relapse occurred in the second month of ruxolitinib treatment in a patient diagnosed with AML, while the other occurred in the twenty-third month of treatment in a patient with ALL. We did not consider the AML relapse to be treatment-related, as it occurred early during ruxolitinib treatment. Wu et al.^27^ reported a relapse rate of 14.6% in their study, while Zeiser et al.^14^ reported a low incidence of disease relapse (2.4%) during ruxolitinib treatment. Similarly, Ferreira et al.^16^ observed a low relapse rate (6%). Based on the findings of this study, it appears that ruxolitinib treatment does not increase the risk of disease relapse. However, it is imperative to emphasize the need for further studies with prolonged follow-up periods similar to validate these findings.

Cytopenias were observed as the most prevalent treatment-related toxicity (34.8%), with only two patients experiencing Grade ≥3 cytopenias, both of which resolved upon dose reduction. Ferreira et al.^16^ reported a similar general cytopenia rate of 31%, consistent with these findings. Moisev et al.^29^ noted Grade 4 cytopenias in less than 15% of cGvHD patients. Given that JAK-STAT pathways play a crucial role in cytokine-mediated hematopoiesis, it is unsurprising that thrombocytopenia or anemia emerge as common side effects in studies investigating ruxolitinib use.^5^^,^^9^^,^^17^^,^^29^

According to the findings of this study, CMV reactivation was not observed during cGvHD treatment despite a high proportion of donor or recipient CMV seropositivity (78%). Similarly, Dang et al.^15^ did not observe CMV reactivation in their study. In contrast, Zeiser et al.^14^ reported CMV activation rates of up to 14.6% in cGvHD patients, while Modi et al.^20^ observed a lower rate of CMV infection (8.6%). Given reported cases of CMV reactivation, frequent monitoring of CMV copy numbers in patients receiving ruxolitinib treatment remains important.^5^ Within the current cGvHD patient cohort, herpes zoster infections were recorded in 8.7% and COVID-19 infections in 13% of cases. A prior study documented a herpes zoster infection rate of 7.1% in cGvHD patients.^15^ Notably, the heightened COVID-19 infection rate may be attributed to the ongoing COVID-19 pandemic during the observation period.

Regarding bacterial infections, this study observed a lower occurrence rate (13%) than literature reports. Abedin et al.^22^ identified bacterial infections in 21% of cGvHD patients, while Modi et al.^20^ reported a 52% infection rate during ruxolitinib treatment. The relatively low infection rate reported here might be associated with the absence of severe Grade 3–4 neutropenia. Additionally, reducing or discontinuing steroid doses in all patients may have contributed to this outcome. Based on these data, it seems that ruxolitinib treatment does not significantly increase the risk of severe infection.

Examining the biology of cGvHD development reveals a progression through three stages. Initially, cytotoxic tissue damage triggers the activation of innate immune system cells, fibroblasts, and endothelial cells. Subsequently, the adaptive immune system becomes hypersensitive while immune regulators decrease. The final stage is characterized by abnormal tissue repair and fibrosis, driven by activated macrophages producing transforming growth factor beta and platelet-derived growth factors, promoting fibroblast activation.^30^ Ruxolitinib may exhibit greater efficacy during the second phase of disease progression and less efficacy during the fibrosis-dominated third phase. Patient selection could play a pivotal role in enhancing treatment responses. Huravelle et al.^14^ reported that ruxolitinib treatment softened the skin in eight out of 12 patients with a scleroderma pattern of cGvHD but did not reduce the affected skin area. Similarly, Xue et al.^28^ found that ruxolitinib treatment did not yield significant improvement in patients with fasciitis, a sclerotic-type of cGvHD of the skin. Of this cohort, five patients exhibiting severe skin involvement in cGvHD, characterized by notable sclerotic changes and fibrosis, did not respond to ruxolitinib treatment, potentially attributable to the advanced stage of their cGvHD.

Limitations of this study include the small patient cohort and its retrospective nature.

This study underscores ruxolitinib as an effective and safe salvage treatment option for cGvHD patients, evidenced by an ORR of 78.3% and a high CR rate of 72.2% of the responders. Given the often prolonged duration of cGvHD treatment, assessing the long-term sustainability of response and potential consequences of ruxolitinib therapy is crucial. As the number of long-term follow-up studies increases, the impact of this treatment on cGvHD will become more evident. However, prospective multicenter studies are merited in confirming our findings.

## Author contribution

Neslihan Mandaci Sanli: Conceptualization, Methodology, Formal analysis, Investigation, Data curation, Writing - Original Draft, Writing - Review & Editing, Supervision, Project administration. Esen Karakuş: Investigation, Data curation, Resources, Writing - Review & Editing, Visualization.

## Conflicts of interest

The authors declare no conflicts of interest.
